# Reprogramming Cells for Brain Repair

**DOI:** 10.3390/brainsci3031215

**Published:** 2013-08-06

**Authors:** Alyx T. Guarino, Randall D. McKinnon

**Affiliations:** Neurosurgery, Rutgers—Robert Wood Johnson Medical School, 125 Patterson St. CAB 7084, New Brunswick, NJ 08903, USA; E-Mail: aguarino@eden.rutgers.edu

**Keywords:** myelin, repair, reprogramming, transplants

## Abstract

At present there are no clinical therapies that can repair traumatic brain injury, spinal cord injury or degenerative brain disease. While redundancy and rewiring of surviving circuits can recover some lost function, the brain and spinal column lack sufficient endogenous stem cells to replace lost neurons or their supporting glia. In contrast, pre-clinical studies have demonstrated that exogenous transplants can have remarkable efficacy for brain repair in animal models. Mesenchymal stromal cells (MSCs) can provide paracrine factors that repair damage caused by ischemic injury, and oligodendrocyte progenitor cell (OPC) grafts give dramatic functional recovery from spinal cord injury. These studies have progressed to clinical trials, including human embryonic stem cell (hESC)-derived OPCs for spinal cord repair. However, ESC-derived allografts are less than optimal, and we need to identify a more appropriate donor graft population. The cell reprogramming field has developed the ability to trans-differentiate somatic cells into distinct cell types, a technology that has the potential to generate autologous neurons and glia which address the histocompatibility concerns of allografts and the tumorigenicity concerns of ESC-derived grafts. Further clarifying how cell reprogramming works may lead to more efficient direct reprogram approaches, and possibly* in vivo* reprogramming, in order to promote brain and spinal cord repair.

## 1. Introduction

The focal problem for traumatic brain and spinal column injury, or neuro-degenerative disease, is that the CNS, unlike all other epithelial tissues, does not self-repair. Neuronal axons damaged by spinal cord injury do not regenerate, in part because neurite outgrowth is blocked by a glial scar at the injury site and by inhibitors in myelin [1]. Neuronal and glial cell bodies that are damaged or destroyed are also not replaced, in part due to the lack of uncommitted stem/progenitor cells in the adult CNS. There are however two known exceptions—ongoing *de novo* neurogenesis in the hippocampus and olfactory bulb and the limited replacement of myelin forming oligodendrocytes (OL) in multiple sclerosis (MS) lesions. The MS lesion repair is particularly illuminating, as partial remyelination correlates with transient improvement of clinical symptoms in relapse-remitting MS. However, this endogenous repair is ultimately overpowered by the disease process [2,3,4]. These observations suggest that functional repair may be possible if we could enhance the stem/progenitor cell pool at the injury site. This has now proven true using cell transplants (exogenous repair) in many preclinical models of genetic, chemical and traumatic injury, and this opens the door for clinical transplants. This review will briefly summarize the preclinical studies on myelin cell transplants which led to the first clinical trials for stem cell based therapy, then examine the status of cell reprogramming in order to generate autologous cells for brain repair.

## 2. Pre-Clinical Transplants

**Figure 1:**
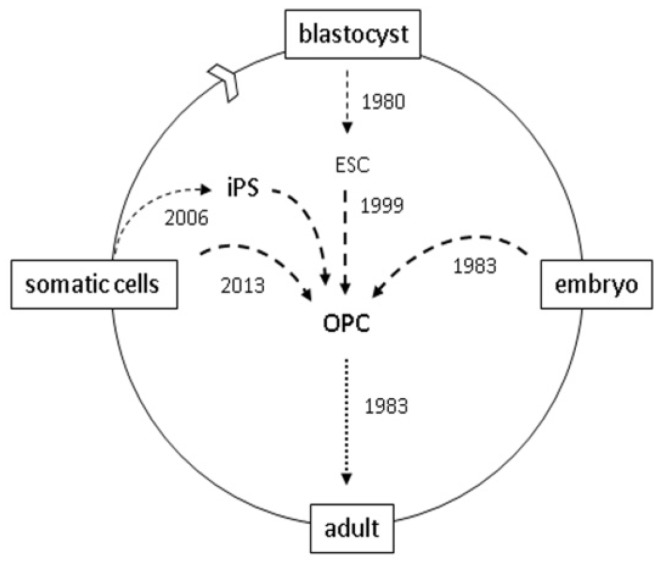
oligodendrocyte progenitor cell (OPC) resources for brain and spinal cord grafts. 1983: OPCs were first characterized in rodents [7]; OPCs were first grafted into shiverer mice [5]; 1999: OPCs generated by* in vitro* differentiation of mouse blastocyst-derived embryonic stem cells (ESCs) [16]; 2005: OPCs used to repair spinal cord injured rats [17]. 2006: human OPCs generated from induced pluripotent stem (iPS) cells [18]; 2010: human ES-derived OPCs first used in clinical trials; 2013: murine OPCs generated by direct cell reprogramming [19,20].

The myelin field pioneered neural cell transplants and demonstrated repair in a variety of animal models including developmental defects, myelin destruction from injury (viral pathogens, chemical toxins), and autoimmune demyelination models of MS (experimental allergic encephalomyelitis). The consensus from these studies is that mitotic and mobile oligodendrocyte progenitor cells (OPCs) are the optimal graft cell population and that immune suppression with steroids or adjunctive MSC is necessary. Early studies from Dr. Gumpel’s group [5] grafted rodent then human brain tissue into *shiverer* (*shi*) mice. Shi mice lack the myelin basic protein gene [6]; their non-compacted CNS myelin degenerates (dys-myelination) and they develop movement-stimulated tremors by two weeks and die at 3–4 months due to sleep apnea. While it does not mimic the autoimmune demyelination of MS, this model facilitates an analysis of the remyelinating ability of transplant derived OLs. Recent advances in this field (Figure 1) came after the characterization of OPCs from the neonatal rat brain [7] and ligands that control OPC proliferation, differentiation and survival* in vitro* [8,9]. This allowed the amplification and transplant of pure OPCs populations [10], identification of the optimal maturation stage for myelination [11] and ultimately the rescue of the Shi motor phenotype and lethality [12,13,14]. The Shi mouse model is now a graft-curable genetic lethal disease, and Shi mice have been used to estimate the minimal number of wild-type cells necessary for functional rescue (7% graft chimerism) [13]. This may represent an upper limit, as co-transplants with adjunctive MSCs improves the survival of transplanted OPCs [15].

## 3. Clinical Transplants

One of the major challenges for transplant-mediated repair is to identify an appropriate graft-competent cell population, as has become evident from two recent clinical trials. The NIH sponsored the first placebo-controlled neurosurgical trial in the US to treat Parkinson’s Disease (PD). The objective was to replace dopaminergic neurons using fetal brain allografts. The pre-graft tissue preparation proved challenging indicating that the grafts need to be optimized and standardized, and the results revealed the need for a reliable index of how many grafted cells are necessary for functional improvement [21,22]. The potential for pluripotent stem cell derived transplants for PD was subsequently demonstrated in animal models [23]. Subsequent clinical trials (Geron Inc., Stem Cell Inc., Neurostem Inc.) are now evaluating the potential of allografts in traumatic spinal cord injury (SCI). In the Geron trial the objective is to use hESC-derived OPCs to reverse the post-trauma loss of OLs caused by neurotransmitters and cytokines in the wound site. Studies that set the stage for this trial (Figure 1) include pioneering work on human ESCs [24] and the pre-clinical demonstration that ESC-derived OPC grafts generate myelin* in vivo* [16] and improve recovery from SCI [17,25] and in Shi mice [26]. 

While stem cell-derived allografts are easier than fetal tissue to standardize, they have safety concerns that will likely limit their utility. Manipulating ESCs in order to generate neural stem/progenitor cells is a long and complex cell culture process. This can expand cells past their “Hayflick limit” [27], conditions that can select for immortalized cells as found with grafted NSCs [28]. ESC-derived cultures can also retain starting ESCs with teratogenic potential [29]. The ideal cells for clinical therapeutics would be ethically acceptable, histo-compatible and non-tumorigenic. Fetal tissue and blastocyst-derived hESC lines fail the first criteria, while existing hESC lines do not satisfy the second and third. An important step toward resolving these limitations emerged with the development of somatic cell reprogramming. Drs. Gurdon and Yamanaka were awarded the 2012 Nobel Prize for their studies on reprogramming of somatic cells into distinct cell types, a critical step toward generating autologous cells for tissue repair.

## 4. Reprogramming

Gurdon and Uehlinger [30] extended the work of Briggs and King [31] on somatic cell nuclear transfer (SCNT) to show that the fertilized, enucleated egg can reprogram donor nuclei from intestinal epithelia cells and generate developmentally normal frogs. This led to the dogma of somatic nuclear equivalence [32], which was extended to mammals by Ian Wilmut in the cloning of Dolly the sheep. This also predicts that the fertilized egg contains cytoplasmic factors which can reprogram somatic nuclei [32,33]. A pivotal clue to the potential identity of those factors came when Yamanaka identified four transcription factors expressed in ESCs (*Oct3/4*, *Sox2*, *Klf4*, *c-Myc*; OSKM) that reprogram fibroblasts into induced pluripotent stem (iPS) cells [18]. They built on prior studies [34] that showed the over expression of a single factor, MyoD, could convert fibroblasts into muscle cells. Thus the Yamanaka factors dictate ESC identity and are analogous to master regulatory genes first proposed by Britten and Davidson [35]. Other combinations of transgenes can also generate iPS cells [36] and at present it is not known whether OSKM are the egg cytoplasm factors that reprogram somatic nuclei during SCNT, or whether they initiate reprogramming by activating other master regulatory genes.

The iPS cells share key features of ES cells including self-renewal, pluripotency and the ability to differentiate into a large number of distinct cell types [37,38].The technology has been extended to human cells [36,39,40] using one factor, Oct4 [41,42], contingent on starting cell type and epigenetic chromatin modifications [43,44,45]. A variety of patient-specific (histocompatible) cells have been used for iPS engineering including embryonic and adult dermal fibroblasts, MSCs and neonatal cord blood. A novel protocol was developed to reprogram renal cells easily harvested from urine [46]. An expanding number of cryo-preservation centers are now banking such cells in order to provide autologous replacement cells lost through trauma or disease. For diseases with a genetic component, the ability to correct a gene defect [47] increases the utility of these resources. In addition to their clinical applications, iPS lines from patients also have the potential for* in vitro* disease modeling and pharmaceutical screens [33].

## 5. Direct Reprogramming

While iPS cells have much potential they may not be true ESC cells, as they can have variable chromatin landscapes [48,49]. However they are equivalent to ESCs in their tumorigenic potential, a risk that could be even greater for autologous grafts, and thus share the same limitations for regenerative medicine. Thus there is currently much interest in identifying methods to convert (*i.e.*, reprogram) patient-specific somatic cell populations directly into other cell types, including neural cells for brain therapy. Early studies on trans-differentiation were controversial [50] and with few exceptions generated partially reprogrammed cells. *In vitro* studies of MSCs reprogrammed into neurons [51] were not corroborated [52], and* in vivo* reports of lineage conversion were misinterpretations due to cell fusion [53,54]. Yamanaka’s pioneering studies have since stimulated a number of groups to re-examine direct reprogramming of somatic cells into defined cell types. To date a number of studies have now demonstrated direct reprogramming, including pancreatic exocrine cells reprogrammed into β-cells and fibroblasts into hepatocyte-like cells, cardiomyocytes, haematopoietic progenitors, neurons and OPCs.

Direct reprogramming requires specific transgenes, and at present the efficiency is low and questions remain about their degree of functionality and long-term stability [55]. Three transcription factors (*Ngn3Pdx1*, *Mafa*) are sufficient to reprogram pancreatic exocrine cells into cells with the morphology, transcriptome and insulin production of β-cells [56]. Similarly, Hnf4 co-expressed with either Foxa1, a2 or a3 is sufficient to convert fibroblasts to hepatocytes that can reconstitute damaged liver [57]. Fibroblasts can also be reprogrammed to cardiomyocytes using Gata4, Mef2c and Tbx5 [58], or by transient induction towards pluripotency (Oct4, Sox2, Klf4) followed by cardiomyocyte differentiation in the presence of small molecule inhibitors [59]. The cells obtained were functionally and morphologically consistent with a cardiomyocyte phenotype. Fibroblasts have also been directly converted to multi-lineage blood progenitors using Oct4 plus cytokines [60], and recent studies report reprogramming of MEFs into apparent myelin forming OPCs using Olig2, Sox10 and either Nkx6.2 or Zfp536 [19,20].

A number of reports also demonstrate the direct conversion of somatic cells into neurons, and again these studies indicate that subsets of transcription factors reprogram fibroblasts into specialized neuronal cell types. Wernig and colleagues [61] demonstrated that three factors (*Ascl1*, *Brn2*, *Myt1l*) can reprogram fibroblasts into induced neuron (iN) cells with the morphology, marker expression, and electrically coupled synapse formation of mature neurons. Most iN were excitatory although a small percentage expressed the inhibitory GABA receptor markers. Subsequent studies demonstrated that combining *Ascl1*, *Brn2* and *Mytrl1* with *Lmx1a* and *FoxA2*, two factors involved in the development of dopaminergic neurons, could reprogram human fibroblasts to dopaminergic neurons [62]. The induced cells were positive for tyrosine hydroxylase, essential for dopamine biosynthesis, and showed action potentials. Fibroblasts can also be reprogrammed into motor neurons (iMN) using *Ascl1*, Brn2 and *Mytl1* plus *Lhx3*, *Hb9*, *Isl1* and *Ngn2* [63]. The iMN express *NeuroD* and Isl1, *ChAT* which is required for acetylcholine synthesis, and exhibit action potentials. Finally, in a unique approach, Berninger and colleagues demonstrated that Mash1 plus *Sox2* can reprogram CNS pericytes into β_3_-tubulin positive neurons, a prelude to what can become a target cell for converting resident brain cells into desired cell types* in situ* [64].

## 6. The Mechanism of Cell Reprogramming

At present we are far from understanding the mechanism of reprogramming. For iPS reprogramming, retroviral gene delivery results in transient OSKM expression followed by activation of the endogenous core ESC gene circuit [38,43]. The slow time course and low frequency suggests a stochastic process [65] consistent with transcriptome sampling [66], rather than a deterministic process with a fixed and predictable start intermediate and end points. Cells that are caught at intermediate stages are not completely reprogrammed [18,65,67,68]. It is also not clear whether culture conditions provide survival (*selection*) or instructive (*induction*) cues. The de-condensation of donor chromatin is an early event in the one known natural reprogramming event (fertilization) and during experimental reprogramming by SCNT [33]. Chromatin modifying proteins also emerged as critical reprogramming factors in *C. elegans* [69], amphibia [70] and mammalian cells [43,45]. Thus global de-condensation may facilitate epigenetic chromatin remodeling during reprogramming.

**Figure 2:**
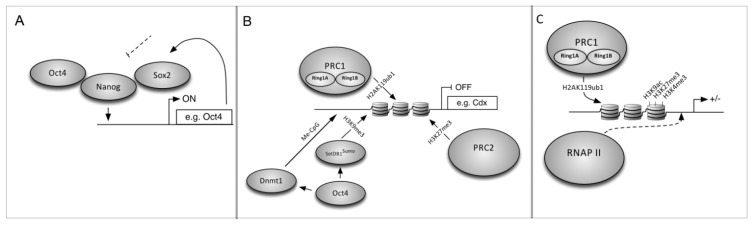
On, off, and poised loci. (**A**) Oct3/4, Sox2 and Nanog positively regulate genes necessary for pluripotency and self renewal in ES cells; (**B**) ES cells also silence genes in order to remain pluripotent; Oct3/4 coordinates CpG DNA methylation and H3K9 histone methylation via DNA methyltransferase and sumoylated SetDB1; (**C**) H2A-K119 ubiquitination by PRC1 is necessary for RNA Polymerase (PolII) to maintain bivalent genes poised for activation.

iPS reprogramming requires suppression of the fibroblast transcriptome and activation of ESC-specific genes, and reprogramming fibroblasts into iPS cells serves as a model for understanding how cell identity is maintained and revised. The ESC master regulators Oct4, Sox2 and Nanog [71,72,73] coordinately regulate 353 known genes to control ESC identity [74]. These include micro-RNAs (miRNAs) that are important for ESC pluripotency and differentiation [75] and have a role in Dicer-targeted destruction of ESC-specific mRNAs during differentiation [76]. They also regulate their own promoters in a positive-feedback loop to promote the undifferentiated state. ESCs differentiate to trophectoderm when Oct4 levels are below 50%, into primitive endoderm and mesoderm when Oct4 is above 150% of wild-type [77], or into endoderm and trophectoderm in the absence of Nanog [78]. These factors bind to *cis*-regulatory DNA sites and recruit chromatin interacting co-factors [79] and RNA Pol II [75] to activate gene expression (Figure 2A). Oct4 can also repress genes (Figure 2B) such as the trophectoderm factor Cdx2 [75,80] which, if expressed, can feed back to repress pluripotency genes. Mechanisms that maintain transcriptionally silent heterochromatin include epigenetic chromatin marks such as DNA methylation and histone modifications (methylation, acetylation) and histone variant exchange.The Oct4 SUMO-interacting motif (SIM) recruits a histone methyltransferase (SUMOylated ESET) which methylates histone H3 on specific lysines (H3K9) to repress Cdx2 expression [80]. Other repressive modifications are generated by the Polycomb Repressive Complex (PRC) [81] including H2A mono-ubiquination by PRC1 and H3 tri-methylation by PRC2 [82]. PRC1 can also maintain a subset of genes in a silenced but actionable “bivalent” state (Figure 2C) and recruit RNA PolII to these genes via H2A ubiquitination [75,83].

A proposed mechanism for iPS reprogramming suggests that ectopic Oct4 and Sox2 serve as pioneer molecules to displace histone octamers at target gene promoters, as found for yeast transcription activators [84]. They then recruit chromatin remodeling complexes and transcription factors to globally alter the transcriptome identity and establish the ESC-specific transcriptome, which in turn suppresses fibroblast-specific genes [85,86]. Individual roles for OSKM factors are starting to be revealed [87,88]. For example, **Sox2** induces Sox21 which represses the endoderm cell fate regulator Cdx2 [89]. Sox2 is stabilized when phosphorylated (Thr118) by Akt, and OSKM vectors have lower iPS induction with mutant (Sox^T118A^) compared to wild-type Sox2 [90]. **Oct4** and Nanogupregulate DNA cytosine-5-methyl transferase (Dnmt1), suggesting Dnmt1-mediated CpG methylation silences differentiation-related genes in ESCs [91,92]. It has been known for quite some time that fibroblasts could be reprogrammed into myocytes and adipocytes when treated with 5-azacytidine to inhibit DNA methylation, again suggesting chromatin modifications play an important role [55,93]. Finally, the proto-oncogenes **Klf4** and **c-Myc** may promote self-renewal to fix epigenetic chromatin modifications, and c-Myc may promote an autoregulatory loop of endogenous *Oct4*, *Sox2* and *Nanog* [94,95]. However neither is essential [45,87,94] and they can be substituted by mutations in the p53 tumor suppressor protein [96,97] which phenocopy proto-oncogenes to promote cell proliferation. Pluripotency can be induced with Oct4 alone in cells that express other necessary factors such as adult neural stem cells, neonatal epidermal keratinocytes and hair follicle dermal papilla [42,91,98]. In HEKs, Oct4 induces pluripotency when combined with a cocktail of small molecules that target regulators and signaling pathways [42].

A novel approach to reprogram fibroblasts into neurons came from the analysis of post-transcriptional gene silencing during the decision for neural progenitor cells to either proliferate or differentiate [99]. The Swi/Snf-like BAF chromatin remodeling complex contains BAF53b, which is essential for neurogenesis, and BAF53a which inhibits neurite outgrowth by antagonizing BAF53b [99]. The miRNAs miR-9/9* and miR-124 bind the 3′ UTR of BAF53a mRNA to repress BAF53a, and they are essential for dendritic morphogenesis. When infected with a lentiviral vector containing miR-9/9* and miR-124, human neonatal foreskin fibroblasts decrease proliferation, express neuronal markers (MAP2) and undergo morphological characteristics within thirty days post-infection. Yoo and colleagues optimized reprogramming by co-expressing miR-9/9* and miR-124 with the neurogenic transcription factors NeuroD2, Ascl1, and Myt1L [100]. These results again demonstrated a clear role for chromatin remodeling complexes and their regulators in the mechanism of cell conversion.

Together these studies are leading to a clearer picture of how pioneer molecules initiate the cell reprogramming process. As this mechanism unfolds, we can anticipate studies which reveal ways to direct this process for targeted reprogramming of specific cells into required cell types [101]. Ultimately this could lead to* in vivo* strategies to reprogram endogenous brain cells, such as brain pericytes [64], into specific types of neurons or glia in order to stimulate brain repair.

## 7. Conclusions

Reprogramming has challenged the dogma of progressive restriction of developmental potential and redefined our notion of cell lineage commitment and plasticity. To date direct reprogramming has generated a variety of brain cell types including tripotent neural stem cells as well as acetylcholine, dopamine and GABA neurons [55]. The recent progress in this field indicates that the two major obstacles, reprogramming efficiency and lineage specificity, will be resolved and it will soon be possible to reprogram readily accessible somatic cells into virtually any cell type. This has tremendous implications for cell and molecular studies of complex disease phenotypes* in vitro*. The genetic correction of identified disease genotypes [47] can also expand clinical options to include transplant based regenerative medicine for tissue repair. While the risk of invasive surgery may limit transplant based brain therapy, the current pace of this field suggests that transgene mediated *in vivo* reprogramming could providea safe alternative to promote brain repair.
